# Butyl 2-(5-iodo-3-methyl­sulfinyl-1-benzofuran-2-yl)acetate

**DOI:** 10.1107/S1600536809000208

**Published:** 2009-01-08

**Authors:** Hong Dae Choi, Pil Ja Seo, Byeng Wha Son, Uk Lee

**Affiliations:** a Department of Chemistry, Dongeui University, San 24 Kaya-dong Busanjin-gu, Busan 614-714, Republic of Korea; b Department of Chemistry, Pukyong National University, 599-1 Daeyeon 3-dong Nam-gu, Busan 608-737, Republic of Korea

## Abstract

In the title compound, C_15_H_17_IO_4_S, the O atom and the methyl group of the methyl­sulfinyl substituent lie on opposite sides of the plane of the benzofuran fragment. The crystal structure is stabilized by weak inter­molecular C—H⋯π inter­actions between a methyl H atom of the methyl­sulfinyl group and the benzene ring of the benzofuran system, and by an I⋯O halogen bond of 3.173 (3) Å and a nearly linear C—I⋯O angle of 171.7 (1)°. In addition, the crystal structure exhibits weak inter­molecular C—H⋯O hydrogen bonds. The O atom of the carbonyl group and the butyl chain are both disordered over two positions with site-occupancy factors from refinement of 0.55 (4) and 0.45 (4) (for the O atom), and 0.76 (2) and 0.24 (2) (for the butyl group).

## Related literature

For the crystal structures of similar alkyl 2-(5-iodo-3-methyl­sulfinyl-1-benzofuran-2-yl)acetate derivatives. see: Choi *et al.* (2007 ▶, 2008 ▶). For a review of halogen bonding, see: Politzer *et al.* (2007 ▶).
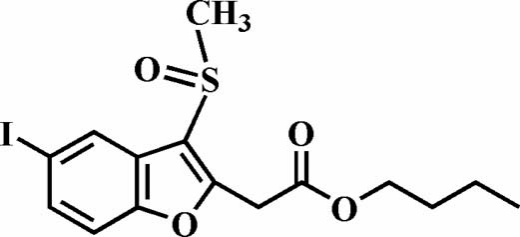


## Experimental

### 

#### Crystal data

C_15_H_17_IO_4_S
*M* *_r_* = 420.25
Monoclinic, 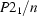

*a* = 10.298 (1) Å
*b* = 15.208 (1) Å
*c* = 11.109 (1) Å
β = 100.669 (1)°
*V* = 1709.7 (3) Å^3^
*Z* = 4
Mo *K*α radiation
μ = 2.01 mm^−1^
*T* = 100 (2) K
0.20 × 0.20 × 0.10 mm

#### Data collection

Bruker SMART CCD diffractometer
Absorption correction: multi-scan (*SADABS*; Sheldrick, 1999 ▶) *T*_min_ = 0.673, *T*_max_ = 0.822
8829 measured reflections
3009 independent reflections
2581 reflections with *I* > 2σ(*I*)
*R*_int_ = 0.017

#### Refinement

*R*[*F* ^2^ > 2σ(*F* ^2^)] = 0.032
*wR*(*F* ^2^) = 0.085
*S* = 1.07
3009 reflections
222 parameters
43 restraints
H-atom parameters constrained
Δρ_max_ = 0.71 e Å^−3^
Δρ_min_ = −0.61 e Å^−3^

### 

Data collection: *SMART* (Bruker, 2001 ▶); cell refinement: *SAINT* (Bruker, 2001 ▶); data reduction: *SAINT*; program(s) used to solve structure: *SHELXS97* (Sheldrick, 2008 ▶); program(s) used to refine structure: *SHELXL97* (Sheldrick, 2008 ▶); molecular graphics: *ORTEP-3* (Farrugia, 1997 ▶) and *DIAMOND* (Brandenburg, 1998 ▶); software used to prepare material for publication: *SHELXL97*.

## Supplementary Material

Crystal structure: contains datablocks global, I. DOI: 10.1107/S1600536809000208/hg2458sup1.cif

Structure factors: contains datablocks I. DOI: 10.1107/S1600536809000208/hg2458Isup2.hkl

Additional supplementary materials:  crystallographic information; 3D view; checkCIF report

## Figures and Tables

**Table 1:** Hydrogen-bond geometry (Å, °)

*D*—H⋯*A*	*D*—H	H⋯*A*	*D*⋯*A*	*D*—H⋯*A*
C15—H15*B*⋯*Cg*^i^	0.98	2.97	3.722 (4)	134
C5—H5⋯O4^ii^	0.95	2.46	3.370 (4)	160
C9—H9*B*⋯O4^iii^	0.99	2.50	3.376 (4)	147

Symmetry codes: (i) 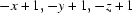; (ii) 
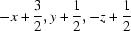
; (iii) 
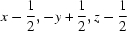
. *Cg* is the centroid of the C2–C7 benzene ring.

**Fig. 1.**
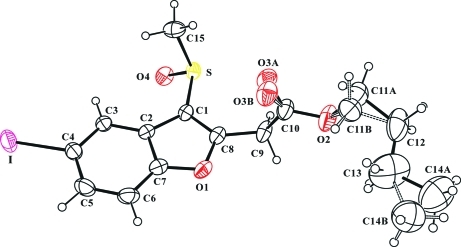
The molecular structure of the title compound, showing displacement ellipsoids drawn at the 30% probability level. The oxygen atom of carbonyl group and butyl group are disordered over two positions with site–occupancy factors, from refinement of 0.55 (4) and 0.45 (4) (for the O atom), 0.76 (2) and 0.24 (2) (for the butyl group), respectively.

**Fig. 2.**
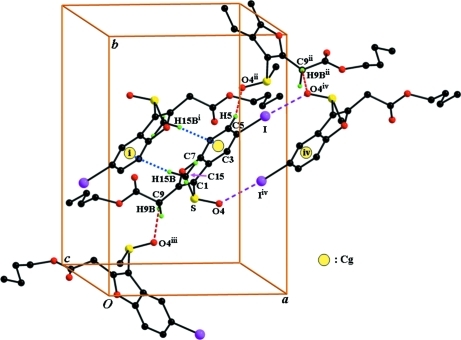
C—H···π, I···O halogen bond and C—H···O interactions (dotted lines) in the title compound. Cg denotes ring centroid. The disordered component of the oxygen atom of carbonyl group and butyl group, part B, have been omitted for clairty as have H atoms not involved in intermolecular contacts. [Symmetry code: (i) -x+1, -y+1, -z+1 ; (ii) x-1/2, -y+1/2, z-1/2; (iii) x-1/2, -y+1/2, z-1/2; (iv) -x+2, -y+1, -z+1.]

## supplementary crystallographic information

### Comment

This work is related to our previous communications on the synthesis and structure of alkyl 2-(5-iodo-3-methylsulfinyl-1-benzofuran-2-yl)acetate analogues, *viz*. ethyl 2-(5-iodo-3-methylsulfinyl-1-benzofuran-2-yl)acetate (Choi *et al.*, 2007) and isopropyl 2-(5-iodo-3-methylsulfinyl-1-benzofuran-2-yl)acetate (Choi *et al.*, 2008). Here we report the crystal structure of the title compound, butyl 2-(5-iodo-3-methylsulfinyl-1-benzofuran-2-yl)acetate (Fig. 1).

The benzofuran unit is essentially planar, with a mean deviation of 0.004 (3) Å from the least-squares plane defined by the nine constituent atoms. The oxygen atom of carbonyl group is disordered over two positions with site–occupancy factors of 0.55 (4) (for atom labelled A) and 0.45 (4) (for atom labelled B), and the butyl group over two positions with site–occupancy factors of 0.76 (2) (for atom labelled A) and 0.24 (2) (for atom labelled B), respectively, in Fig. 1. The molecular packing (Fig. 2) is stabilized by intermolecular C—H···π interactions between a methyl H atom of the methylsulfinyl group and the benzene ring of the benzofuran unit, with a C15—H15B···*Cg*^i^ separation of 2.97 Å (Table 1 and Fig. 2; *Cg* is the centroid of the C2–C7 benzene ring, symmetry code as in Fig. 2). The molecular packing is further stabilized by an I···O halogen bond (Politzer *et al.*, 2007) between the iodine atom and the oxygen of a neighbouring S═O unit, with an I···O4^iv^ distance of 3.173 (3) Å (symmetry code as in Fig. 2). In addition, weak intermolecular C—H···O hydrogen bonds in the structure are observed (Table 1 & Fig. 2).

### Experimental

77% 3-chloroperoxybenzoic acid (123 mg, 0.55 mmol) was added in small portions to a stirred solution of butyl 2-(5-iodo-3-methylsulfanyl-1-benzofuran-2-yl)acetate (202 mg, 0.5 mmol) in dichloromethane (30 ml) at 273 K. After being stirred for 3 h at room temperature, the mixture was washed with saturated sodium bicarbonate solution and the organic layer was separated, dried over magnesium sulfate, filtered and concentrated in vacuum. The residue was purified by column chromatography (hexane-ethyl acetate, 1:2 v/v) to afford the title compound as a colorless solid [yield 80%, m.p. 407-408 K; *R*_f_ = 0.54 (hexane-ethyl acetate, 1;2 v/v)]. Single crystals suitable for X-ray diffraction were prepared by evaporation of a solution of the title compound in acetone at room temperature. Spectroscopic analysis: ^1^H NMR (CDCl_3_, 400 MHz) δ 0.92 (t, J = 7.68 Hz, 3H), 1.31-1.42 (m, 2H),1.59-1.67 (m, 2H), 3.07 (s, 3H), 4.03 (s, 2H), 4.15 (t, J = 6.6 Hz, 2H), 7.29 (d, J = 8.8 Hz, 1H), 7.66 (dd, J = 8.8 Hz and J = 1.84 Hz, 1H), 8.29 (d, J = 1.84 Hz, 1H); EI-MS 420 [M^+^].

### Refinement

All H atoms were geometrically positioned and refined using a riding model, with C—H = 0.95 Å for the aryl, 0.99 Å for the methylene, and 0.98 Å for the methyl H atoms. Uiso(H) = 1.2*U*eq(C) for the aryl and methylene H atoms, and 1.5*U*eq(C) for methyl H atoms. The oxygen atom of carbonyl group and butyl group were found to be disordered over two positions and modelled with site-occupancy factors, from refinement of 0.55 (4) (O3A) and 0.45 (4) (O3B), and 0.76 (2) (C11A–C14A)) and 0.24 (2) (C11B–C14B), respectively. The displacement ellipsoids of part B were restrained using command ISOR (0.01), both sets of O and C atoms were restrained using the command DELU and the distances of C—C were restrained to 1.480 (2) Å using command DFIX. The distances of C═O were restrained to 0.001 Å using command SADI.

### Crystal data

**Table:**

C_15_H_17_IO_4_S	*F*(000) = 832
*M**_r_* = 420.25	*D*_x_ = 1.633 Mg m^−^^3^
Monoclinic, *P*2_1_/*n*	Mo *K*α radiation, λ = 0.71073 Å
Hall symbol: -P_2yn	Cell parameters from 5617 reflections
*a* = 10.298 (1) Å	θ = 2.3–28.1°
*b* = 15.208 (1) Å	µ = 2.01 mm^−^^1^
*c* = 11.109 (1) Å	*T* = 100 K
β = 100.669 (1)°	Block, colorless
*V* = 1709.7 (3) Å^3^	0.20 × 0.20 × 0.10 mm
*Z* = 4	

### Data collection

**Table:**

Bruker SMART CCD diffractometer	3009 independent reflections
Radiation source: fine-focus sealed tube	2581 reflections with *I* > 2σ(*I*)
graphite	*R*_int_ = 0.017
Detector resolution: 10.0 pixels mm^-1^	θ_max_ = 25.0°, θ_min_ = 2.3°
φ and ω scans	*h* = −12→12
Absorption correction: multi-scan (*SADABS*; Sheldrick, 1999)	*k* = −11→18
*T*_min_ = 0.673, *T*_max_ = 0.822	*l* = −13→13
8829 measured reflections	

### Refinement

**Table:**

Refinement on *F*^2^	Primary atom site location: structure-invariant direct methods
Least-squares matrix: full	Secondary atom site location: difference Fourier map
*R*[*F*^2^ > 2σ(*F*^2^)] = 0.032	Hydrogen site location: difference Fourier map
*wR*(*F*^2^) = 0.085	H-atom parameters constrained
*S* = 1.07	*w* = 1/[σ^2^(*F*_o_^2^) + (0.0408*P*)^2^ + 1.6213*P*] where *P* = (*F*_o_^2^ + 2*F*_c_^2^)/3
3009 reflections	(Δ/σ)_max_ < 0.001
222 parameters	Δρ_max_ = 0.71 e Å^−^^3^
43 restraints	Δρ_min_ = −0.61 e Å^−^^3^

### Special details

**Table:**

Geometry. All esds (except the esd in the dihedral angle between two l.s. planes) are estimated using the full covariance matrix. The cell esds are taken into account individually in the estimation of esds in distances, angles and torsion angles; correlations between esds in cell parameters are only used when they are defined by crystal symmetry. An approximate (isotropic) treatment of cell esds is used for estimating esds involving l.s. planes.
Refinement. Refinement of F^2^ against ALL reflections. The weighted R-factor wR and goodness of fit S are based on F^2^, conventional R-factors R are based on F, with F set to zero for negative F^2^. The threshold expression of F^2^ > 2sigma(F^2^) is used only for calculating R-factors(gt) etc. and is not relevant to the choice of reflections for refinement. R-factors based on F^2^ are statistically about twice as large as those based on F, and R- factors based on ALL data will be even larger.

### Fractional atomic coordinates and isotropic or equivalent isotropic displacement parameters (Å^2^)

**Table:**

	*x*	*y*	*z*	*U*_iso_*/*U*_eq_	Occ. (<1)
I	0.98000 (3)	0.639217 (19)	0.38449 (3)	0.07282 (14)	
S	0.60101 (8)	0.31517 (5)	0.45617 (8)	0.0460 (2)	
O1	0.4557 (2)	0.45536 (17)	0.1655 (2)	0.0562 (6)	
O2	0.1138 (3)	0.3286 (2)	0.2161 (3)	0.0849 (10)	
O3A	0.2384 (15)	0.4110 (13)	0.3619 (13)	0.074 (3)	0.55 (4)
O3B	0.2198 (16)	0.4365 (10)	0.323 (2)	0.075 (4)	0.45 (4)
O4	0.7434 (2)	0.29309 (16)	0.4707 (2)	0.0588 (6)	
C1	0.5648 (3)	0.3928 (2)	0.3367 (3)	0.0411 (7)	
C2	0.6417 (3)	0.4674 (2)	0.3106 (3)	0.0404 (7)	
C3	0.7603 (3)	0.5070 (2)	0.3653 (3)	0.0438 (7)	
H3	0.8119	0.4843	0.4384	0.053*	
C4	0.7992 (3)	0.5803 (2)	0.3085 (3)	0.0520 (8)	
C5	0.7244 (4)	0.6150 (3)	0.2018 (4)	0.0651 (10)	
H5	0.7551	0.6657	0.1658	0.078*	
C6	0.6077 (4)	0.5773 (3)	0.1483 (3)	0.0628 (10)	
H6	0.5555	0.6009	0.0760	0.075*	
C7	0.5693 (3)	0.5030 (2)	0.2046 (3)	0.0492 (8)	
C8	0.4576 (3)	0.3885 (2)	0.2479 (3)	0.0474 (8)	
C9	0.3408 (3)	0.3292 (3)	0.2250 (4)	0.0581 (9)	
H9A	0.3645	0.2724	0.2668	0.070*	
H9B	0.3165	0.3176	0.1360	0.070*	
C10	0.2236 (4)	0.3677 (3)	0.2695 (4)	0.0609 (10)	
C12	−0.1176 (4)	0.3369 (5)	0.1516 (6)	0.121 (2)	
H12A	−0.1186	0.2744	0.1266	0.145*	0.759 (19)
H12B	−0.2018	0.3484	0.1796	0.145*	0.759 (19)
H12C	−0.0930	0.2748	0.1415	0.145*	0.241 (19)
H12D	−0.2016	0.3407	0.1825	0.145*	0.241 (19)
C13	−0.1166 (8)	0.3909 (7)	0.0415 (9)	0.180 (4)	
H13A	−0.0364	0.3789	0.0069	0.216*	0.759 (19)
H13B	−0.1186	0.4543	0.0614	0.216*	0.759 (19)
H13C	−0.0345	0.3687	0.0178	0.216*	0.241 (19)
H13D	−0.0905	0.4483	0.0808	0.216*	0.241 (19)
C11A	−0.0090 (6)	0.3482 (8)	0.2583 (7)	0.077 (2)	0.759 (19)
H11A	−0.0077	0.4093	0.2894	0.092*	0.759 (19)
H11B	−0.0208	0.3077	0.3251	0.092*	0.759 (19)
C14A	−0.2370 (12)	0.3655 (11)	−0.0466 (14)	0.175 (6)	0.759 (19)
H14A	−0.3089	0.4062	−0.0392	0.263*	0.759 (19)
H14B	−0.2627	0.3056	−0.0286	0.263*	0.759 (19)
H14C	−0.2191	0.3679	−0.1301	0.263*	0.759 (19)
C11B	−0.0098 (17)	0.3900 (18)	0.223 (3)	0.077 (7)	0.241 (19)
H11C	−0.0222	0.3983	0.3089	0.093*	0.241 (19)
H11D	−0.0017	0.4480	0.1851	0.093*	0.241 (19)
C14B	−0.185 (4)	0.424 (3)	−0.0790 (17)	0.141 (14)	0.241 (19)
H14D	−0.2681	0.3920	−0.1041	0.211*	0.241 (19)
H14E	−0.1284	0.4149	−0.1399	0.211*	0.241 (19)
H14F	−0.2034	0.4869	−0.0728	0.211*	0.241 (19)
C15	0.5826 (5)	0.3860 (3)	0.5804 (3)	0.0654 (10)	
H15A	0.6071	0.3541	0.6579	0.098*	
H15B	0.4904	0.4053	0.5705	0.098*	
H15C	0.6400	0.4374	0.5807	0.098*	

### Atomic displacement parameters (Å^2^)

**Table:**

	*U*^11^	*U*^22^	*U*^33^	*U*^12^	*U*^13^	*U*^23^
I	0.06107 (19)	0.0672 (2)	0.0881 (2)	−0.02167 (12)	0.00823 (14)	0.01316 (14)
S	0.0464 (4)	0.0356 (4)	0.0542 (5)	−0.0007 (3)	0.0051 (4)	−0.0015 (3)
O1	0.0494 (13)	0.0693 (16)	0.0437 (13)	0.0025 (12)	−0.0075 (10)	−0.0056 (12)
O2	0.0446 (14)	0.110 (2)	0.097 (2)	−0.0015 (15)	0.0058 (14)	−0.053 (2)
O3A	0.072 (5)	0.087 (6)	0.064 (5)	−0.013 (5)	0.016 (4)	−0.030 (4)
O3B	0.069 (5)	0.068 (5)	0.088 (7)	0.000 (4)	0.014 (5)	−0.030 (5)
O4	0.0513 (14)	0.0568 (15)	0.0648 (15)	0.0124 (11)	0.0019 (11)	0.0010 (12)
C1	0.0395 (16)	0.0378 (15)	0.0440 (17)	0.0028 (13)	0.0030 (13)	−0.0046 (13)
C2	0.0405 (16)	0.0447 (17)	0.0350 (15)	0.0049 (13)	0.0045 (12)	−0.0031 (13)
C3	0.0431 (17)	0.0464 (18)	0.0406 (16)	0.0009 (14)	0.0044 (13)	0.0022 (14)
C4	0.0506 (19)	0.052 (2)	0.053 (2)	−0.0045 (16)	0.0100 (15)	0.0052 (16)
C5	0.075 (3)	0.062 (2)	0.060 (2)	−0.003 (2)	0.015 (2)	0.0218 (19)
C6	0.069 (2)	0.075 (3)	0.0423 (19)	0.006 (2)	0.0048 (17)	0.0150 (18)
C7	0.0499 (19)	0.057 (2)	0.0394 (17)	0.0066 (16)	0.0057 (14)	−0.0018 (15)
C8	0.0426 (17)	0.0495 (18)	0.0475 (18)	0.0035 (14)	0.0013 (14)	−0.0100 (15)
C9	0.0427 (18)	0.057 (2)	0.069 (2)	0.0006 (16)	−0.0049 (16)	−0.0229 (18)
C10	0.051 (2)	0.065 (2)	0.066 (2)	−0.0061 (17)	0.0064 (18)	−0.0211 (19)
C12	0.053 (3)	0.160 (6)	0.149 (6)	0.002 (3)	0.021 (3)	−0.065 (5)
C13	0.152 (8)	0.215 (10)	0.166 (9)	0.103 (8)	0.010 (7)	0.029 (8)
C11A	0.049 (3)	0.095 (6)	0.087 (4)	0.004 (3)	0.016 (3)	−0.018 (4)
C14A	0.148 (8)	0.195 (10)	0.167 (9)	0.043 (7)	−0.010 (7)	−0.002 (7)
C11B	0.064 (9)	0.088 (11)	0.084 (11)	0.011 (8)	0.023 (8)	−0.007 (8)
C14B	0.142 (16)	0.142 (16)	0.139 (16)	0.015 (10)	0.027 (10)	0.012 (9)
C15	0.090 (3)	0.058 (2)	0.051 (2)	0.010 (2)	0.022 (2)	−0.0034 (18)

### Geometric parameters (Å, °)

**Table:**

I—C4	2.098 (4)	C9—H9B	0.9900
I—O4^i^	13.531 (3)	C12—C13	1.476 (11)
S—O4	1.484 (2)	C12—C11B	1.480 (2)
S—C1	1.764 (3)	C12—C11A	1.481 (2)
S—C15	1.788 (4)	C12—H12A	0.9900
O1—C8	1.366 (4)	C12—H12B	0.9900
O1—C7	1.376 (4)	C12—H12C	0.9900
O2—C10	1.317 (5)	C12—H12D	0.9900
O2—C11A	1.459 (7)	C13—C14B	1.4797 (15)
O2—C11B	1.59 (3)	C13—C14A	1.481 (2)
O3A—O3B	0.590 (16)	C13—H13A	0.9900
O3A—C10	1.205 (5)	C13—H13B	0.9900
O3B—C10	1.205 (5)	C13—H13C	0.9900
C1—C8	1.339 (4)	C13—H13D	0.9900
C1—C2	1.444 (4)	C11A—H11A	0.9900
C2—C7	1.383 (4)	C11A—H11B	0.9900
C2—C3	1.395 (4)	C14A—H14A	0.9800
C3—C4	1.377 (5)	C14A—H14B	0.9800
C3—H3	0.9500	C14A—H14C	0.9800
C4—C5	1.392 (5)	C11B—H11C	0.9900
C5—C6	1.364 (6)	C11B—H11D	0.9900
C5—H5	0.9500	C14B—H14D	0.9800
C6—C7	1.384 (5)	C14B—H14E	0.9800
C6—H6	0.9500	C14B—H14F	0.9800
C8—C9	1.486 (5)	C15—H15A	0.9800
C9—C10	1.505 (5)	C15—H15B	0.9800
C9—H9A	0.9900	C15—H15C	0.9800
			
C4—I—O4^i^	52.14 (10)	C11B—C12—H12C	113.6
O4—S—C1	107.46 (15)	C11A—C12—H12C	91.8
O4—S—C15	107.26 (19)	H12B—C12—H12C	117.5
C1—S—C15	98.03 (17)	C13—C12—H12D	113.3
C8—O1—C7	106.0 (2)	C11B—C12—H12D	113.4
C10—O2—C11A	119.1 (4)	C11A—C12—H12D	107.1
C10—O2—C11B	109.9 (4)	H12A—C12—H12D	100.9
O3B—O3A—C10	75.8 (4)	H12C—C12—H12D	110.7
O3A—O3B—C10	75.8 (4)	C12—C13—C14B	149.8 (19)
C8—C1—C2	107.4 (3)	C12—C13—C14A	105.6 (10)
C8—C1—S	123.5 (3)	C12—C13—H13A	110.6
C2—C1—S	129.1 (2)	C14B—C13—H13A	90.2
C7—C2—C3	119.5 (3)	C14A—C13—H13A	110.6
C7—C2—C1	104.4 (3)	C12—C13—H13B	110.6
C3—C2—C1	136.0 (3)	C14B—C13—H13B	81.2
C4—C3—C2	117.1 (3)	C14A—C13—H13B	110.6
C4—C3—H3	121.5	H13A—C13—H13B	108.8
C2—C3—H3	121.5	C12—C13—H13C	99.6
C3—C4—C5	122.3 (3)	C14B—C13—H13C	99.4
C3—C4—I	118.3 (2)	C14A—C13—H13C	112.8
C5—C4—I	119.4 (3)	H13B—C13—H13C	116.6
C6—C5—C4	121.1 (4)	C12—C13—H13D	99.6
C6—C5—H5	119.4	C14B—C13—H13D	98.4
C4—C5—H5	119.4	C14A—C13—H13D	130.3
C5—C6—C7	116.6 (3)	H13A—C13—H13D	99.0
C5—C6—H6	121.7	H13C—C13—H13D	104.1
C7—C6—H6	121.7	O2—C11A—C12	106.9 (5)
O1—C7—C2	110.8 (3)	O2—C11A—H11A	110.3
O1—C7—C6	125.8 (3)	C12—C11A—H11A	110.3
C2—C7—C6	123.4 (3)	O2—C11A—H11B	110.3
C1—C8—O1	111.4 (3)	C12—C11A—H11B	110.3
C1—C8—C9	133.4 (3)	H11A—C11A—H11B	108.6
O1—C8—C9	115.2 (3)	C13—C14A—H14A	109.5
C8—C9—C10	112.4 (3)	C13—C14A—H14B	109.5
C8—C9—H9A	109.1	C13—C14A—H14C	109.5
C10—C9—H9A	109.1	C12—C11B—O2	100.4 (12)
C8—C9—H9B	109.1	C12—C11B—H11C	111.7
C10—C9—H9B	109.1	O2—C11B—H11C	111.7
H9A—C9—H9B	107.9	C12—C11B—H11D	111.7
O3A—C10—O2	126.3 (7)	O2—C11B—H11D	111.7
O3B—C10—O2	120.6 (9)	H11C—C11B—H11D	109.5
O3A—C10—C9	120.6 (8)	C13—C14B—H14D	109.5
O3B—C10—C9	126.7 (9)	C13—C14B—H14E	109.5
O2—C10—C9	110.5 (3)	H14D—C14B—H14E	109.5
C13—C12—C11B	91.3 (16)	C13—C14B—H14F	109.5
C13—C12—C11A	118.6 (8)	H14D—C14B—H14F	109.5
C13—C12—H12A	107.7	H14E—C14B—H14F	109.5
C11B—C12—H12A	130.0	S—C15—H15A	109.5
C11A—C12—H12A	107.7	S—C15—H15B	109.5
C13—C12—H12B	107.7	H15A—C15—H15B	109.5
C11B—C12—H12B	110.4	S—C15—H15C	109.5
C11A—C12—H12B	107.7	H15A—C15—H15C	109.5
H12A—C12—H12B	107.1	H15B—C15—H15C	109.5
C13—C12—H12C	113.4		
			
O4—S—C1—C8	135.5 (3)	C7—O1—C8—C1	0.9 (4)
C15—S—C1—C8	−113.5 (3)	C7—O1—C8—C9	178.5 (3)
O4—S—C1—C2	−41.2 (3)	C1—C8—C9—C10	96.8 (5)
C15—S—C1—C2	69.8 (3)	O1—C8—C9—C10	−80.1 (4)
C8—C1—C2—C7	0.7 (3)	O3B—O3A—C10—O2	−88 (3)
S—C1—C2—C7	177.8 (3)	O3B—O3A—C10—C9	112 (3)
C8—C1—C2—C3	179.9 (4)	O3A—O3B—C10—O2	111 (3)
S—C1—C2—C3	−3.0 (5)	O3A—O3B—C10—C9	−87 (3)
C7—C2—C3—C4	−0.5 (4)	C11A—O2—C10—O3A	10.1 (16)
C1—C2—C3—C4	−179.6 (3)	C11B—O2—C10—O3A	39 (2)
C2—C3—C4—C5	0.5 (5)	C11A—O2—C10—O3B	−23.3 (17)
C2—C3—C4—I	−178.0 (2)	C11B—O2—C10—O3B	6(2)
O4^i^—I—C4—C3	55.4 (2)	C11A—O2—C10—C9	172.1 (6)
O4^i^—I—C4—C5	−123.1 (4)	C11B—O2—C10—C9	−159.0 (14)
C3—C4—C5—C6	0.1 (6)	C8—C9—C10—O3A	−36.2 (13)
I—C4—C5—C6	178.6 (3)	C8—C9—C10—O3B	−2.8 (18)
C4—C5—C6—C7	−0.7 (6)	C8—C9—C10—O2	160.6 (4)
C8—O1—C7—C2	−0.4 (4)	C11B—C12—C13—C14B	−158 (4)
C8—O1—C7—C6	−179.7 (3)	C11A—C12—C13—C14B	−168 (3)
C3—C2—C7—O1	−179.6 (3)	C11B—C12—C13—C14A	−168.6 (12)
C1—C2—C7—O1	−0.2 (3)	C11A—C12—C13—C14A	−179.2 (8)
C3—C2—C7—C6	−0.2 (5)	C10—O2—C11A—C12	150.7 (6)
C1—C2—C7—C6	179.1 (3)	C11B—O2—C11A—C12	73.5 (9)
C5—C6—C7—O1	−180.0 (3)	C13—C12—C11A—O2	−59.2 (10)
C5—C6—C7—C2	0.8 (6)	C11B—C12—C11A—O2	−81 (2)
C2—C1—C8—O1	−1.0 (4)	C13—C12—C11B—O2	−98.9 (16)
S—C1—C8—O1	−178.3 (2)	C11A—C12—C11B—O2	62 (2)
C2—C1—C8—C9	−178.0 (3)	C10—O2—C11B—C12	176.1 (12)
S—C1—C8—C9	4.6 (5)	C11A—O2—C11B—C12	−69.0 (8)

Symmetry codes: (i) −*x*+3/2, *y*−1/2, −*z*+1/2.

### Hydrogen-bond geometry (Å, °)

**Table:**

*D*—H···*A*	*D*—H	H···*A*	*D*···*A*	*D*—H···*A*
C15—H15B···Cg^ii^	0.98	2.97	3.722 (4)	134
C5—H5···O4^iii^	0.95	2.46	3.370 (4)	160
C9—H9B···O4^iv^	0.99	2.50	3.376 (4)	147

Symmetry codes: (ii) −*x*+1, −*y*+1, −*z*+1; (iii) −*x*+3/2, *y*+1/2, −*z*+1/2; (iv) *x*−1/2, −*y*+1/2, *z*−1/2.
